# Enrichment of Cellulosic Waste Hemp (*Cannabis sativa*) Hurd into Non-Toxic Microfibres

**DOI:** 10.3390/ma9070562

**Published:** 2016-07-11

**Authors:** Reinu E. Abraham, Cynthia S. Wong, Munish Puri

**Affiliations:** 1 Centre for Chemistry and Biotechnology, School of Life and Environment Science, Deakin University, Waurn Ponds, Geelong 3217, Australia; r.abraham@deakin.edu.au; 2 Institute for Frontier Materials, Geelong Technology Precinct, Deakin University, Geelong 3217, Australia; cynthia.wong@deakin.edu.au; 3 Bioprocessing Laboratory, CCB, Deakin University, Geelong3217, Australia

**Keywords:** alkaline treatment, cellulose, human fibroblasts, scaffolds, valuables

## Abstract

In this study a largely available lignocellulose feedstock hemp (*Cannabis sativa*), obtained as an industrial waste, was used for cellulose extraction. The extraction of cellulose microfibres from hemp biomass was conducted by alkaline treatment and an acidification process. The extracted cellulose microfibres were characterised using Fourier-transformed infrared spectroscopy (FTIR), Scanning electron microscopy (SEM), thermogravimetric analysis (TGA) and X-ray diffraction (XRD). The viability of the study was determined by growing human fibroblasts on the preparation which resulted in being non-toxic; indicating its potential in preparing biological scaffolds. Upon enzymatic hydrolysis of the cellulose microfibre using cellulase from *Trichoderma reesei*, a maximum of 909 mg/g of reducing sugars were obtained, which endorses its suitability for biofuel production.

## 1. Introduction

Lignocellulose is available abundantly as agricultural and industrial waste, feedstock, and woody biomass. The complexity in its structure is due to the cross-linkage of different components such as cellulose, hemicelluloses, lignin and pectin [1]. A large amount of cellulosic waste is generated from industries such as textile and fibre, paper and pulp. These cellulosic residues can be utilised for the production of high-value-added products including biofuel and bioproducts.

Cellulosic wastes can also be utilised for extracting microfibres and nanocrystals, which has good demand in nano/biomaterial due to exceptional mechanical properties, high aspect ratio, higher crystallinity and thermal stability, and large surface area [2,3,4]. Their major applications in nanocomposites include the production of nanocomposite materials, nanotubes and thin films [5,6]; and the properties can be changed to alter its solubility, dispersibility and thermal properties [7]. Studies have reported that bionanocomposites from lignocellulosic residues can find a potential future in biomedical field due to biocompatibility properties [8]. A recent study has demonstrated the utilisation of these cellulose and hemicellulose based whiskers for synthesising hydrogels and spun fibres [9,10].

Research on cellulose microfibre extraction has been done largely but studies on its application are remotely investigated. Few extractions studies involving microfibres have been conducted earlier using spruce bark [11], sweet potato [12], rice husk [13] and agro waste biomass [14].

Hemp (*Cannabis sativa*) biomass is a low-cost softwood, which is mainly grown for the industrial and medicinal application [15]. Due to the outer bast fibre, it is well known for its mechanical strength, durability; and therefore has wide industrial application [16]. The inner core of hemp can be utilised as a potential source for the extraction of microfibres as it is usually considered as waste [17,18,19]. Due to its mechanical properties, hemp cellulose microfibres find potential application for ligament or tendon substitute, tissue regeneration or small grafting [20]. In a recent study, it was observed that cellulose extracted from lignocellulosic residues can be utilised for biorefinery application, such as ethanol production [11].

This study documents the extraction of cellulose microfibres from hemp biomass using alkaline and acidification method. The extracted cellulose microfibres were characterised using attenuated total reflectance-infrared absorption spectroscopy (ATR-FTIR), thermogravimetric analysis (TGA), X-ray diffraction (XRD) and scanning electron microscopy (SEM). Furthermore, to our knowledge, this is the first study where the toxicity of hemp microfibres on human fibroblasts was investigated for its suitability for biomedical application. An enzymatic hydrolysis of extracted cellulose for the production of reducing sugars broadened its relevance as a valuable for biorefinery applications.

## 2. Materials and Methods

### 2.1. Materials

The raw material used in this study was the cellulosic waste (hemp hurds) obtained from the inner core of hemp (*Cannabis sativa*) biomass. The hemp hurds used in the study were grown in New South Wales, Australia (S34°34′, E146°12′) and was harvested in March 2009. The material was dried at 70 °C to obtain a constant weight and then milled in a Fritsch Pulverisette 19 Universal Cutting Mill using sieve diameter of 1 mm. The milled hemp was sieved again manually using a mesh size of 300 µm to remove bigger particles and the particle size used for the experiment varied from 300–600 µm. The chemicals used in the extraction process were sodium hydroxide (Sigma-Aldrich, St. Louis, MO, USA), sulphuric acid (98%, AR grade-Merck, Kenilworth, NJ, USA) and hydrogen peroxide (Chem-supply, Gillman, South Australia, Australia). The enzyme hydrolysis of isolated cellulose was achieved using cellulase from *Trichoderma reesei* (Sigma-Aldrich) [15].

### 2.2. Cellulose Extraction

The extraction of cellulose microfibres and its acid hydrolysis from cellulosic residue was adapted from Taixeira et al. [21]. Milled hemp hurd biomass (HHB) (5 g) was sonicated using deionised water (300 mL) at room temperature. Sonication allowed the breakage of strong bonds in biomass structure and the supernatant was separated using Whatman No. 1 filter paper (Sigma-Aldrich, St. Louis, MO, USA). The filtered biomass was made into the slurry at 55 °C using 100 mL solution of sodium hydroxide (NaOH) (5%, *w*/*v*) and hydrogen peroxide (11%, *v*/*v*). This biomass slurry was stirred vigorously for 90 min and then filtered. The alkaline treated/bleached hemp (ATH) biomass was washed until a neutral pH was attained and then the residue was dried at 50 °C to achieve a constant weight. The procedure was repeated again to achieve a product with effective bleaching and discoloration. These cellulose microfibres obtained after bleaching were exposed to acid hydrolysis to obtain fine microfibres. Acid hydrolysed hemp (AHH) fibres were produced by adding 5 g of bleach dried biomass in 100 mL of 6 M sulphuric acid under vigorous stirring for 30 min and the reaction was stopped by adding 500 mL of cold deionised. The slurry was centrifuged at 10,000 rpm for 10 min and washed with deionised water until neutral pH was achieved.

### 2.3. Characterisation Studies

#### 2.3.1. Scanning Electron Microscopy (SEM)

The SEM was conducted to study the changes occurred in the structure of hemp after alkaline and acid hydrolysis. Samples were mounted on an aluminium stub and coated with gold with the help of sputter coater (BAL-TEC SCD 050, Leica Microsystems, Wetzlar, Germany). The SEM imaging was conducted using Zeiss Supra 55 VP (Carl Zeiss AG, Oberkochen, Germany) having a secondary electron (SE2) detector with an accelerating voltage of 5 KV under a magnification ranging from 2 to 30 µm.

#### 2.3.2. Particle Size Analysis

The particle size analysis was done using Malvern mastersizer particle size analyser (Malvern, UK) equipped with hydro 2000 S dispersion unit. The samples were dissolved in water before dispensing into the instrument. The result was analysed using mastersizer 2000 software.

#### 2.3.3. Attenuated Total Reflection—Fourier Transform Infrared (ATR-FTIR)

The ATR-FTIR spectra of raw and extracted cellulose (ATH and AHH) HHB were recorded using Bruker Optik GmbH (Ettlingen, Germany). The detector used in the instrument was deuterated triglycine sulfate (DTGS) with a single-reflection diamond ATR sampling module (Platinum ATR QuickSnap™, Ettlingen, Germany) in a scanning range of 375 to 4000 cm^−1^ and scan resolution of 4 cm^−1^. The results were analysed using OPUS 6.0 suite (Bruker) software.

#### 2.3.4. X-ray Diffraction (XRD)

The crystallinity of the samples was measured using Panalytical XRD (Panalytical XPert PRO MRD XL, Almelo, The Netherlands) at 30 kV and 40 mA. The spectrum consisted of an average of three individual scans with intensity in the 2θ range from 5° to 30°. The crystallinity indices (CrI) of the samples were calculated using intensities of the amorphous and crystalline regions using the below formula [22]:

CrI = (I_002_ − I_am_)/I_002_ × 100 (1)

where I_am_ represents the amorphous region at 2θ = 15° and I_002_ represents the crystallinity area at 2θ = 22°.

#### 2.3.5. Thermogravimetric Analysis (TGA)

The pyrolytic behaviour of raw and extracted cellulose hemp sample was studied using thermogravimetry analysis (TGA) under nitrogen atmosphere at a constant flow rate of 10 mL/min and heating rate of 10 °C/min. The experiment was performed using 5 mg of sample under a temperature range of 30 °C–700 °C using a Netzsch DSC/TGA (Model STA409PC; NETZSCH-GmbH, New South Wales, Australia).

### 2.4. Enzyme Hydrolysis

The enzyme hydrolysis of raw and extracted cellulose (ATH and AHH) HHB was conducted using cellulase from *Trichoderma reesei* (EC 3.2.1.4; 700 units). The hydrolysis experiment was conducted for 72 h using substrate (raw, ATH and AHH) concentration of 2% (*w*/*v*) and 30 FPU of cellulase in sodium citrate buffer, pH 4.8 (0.05 M). The reducing sugars were estimated using dinitrosalicylic acid (DNS) method [23]. The activity of cellulase was determined using filter paper method [24]. One unit of enzyme activity is defined as 1 μmol of glucose liberated per minute of enzyme assay. All experiments were conducted in triplicate reported as mean values plus or minus the standard deviation.

### 2.5. Toxicity Studies

#### 2.5.1. Sterilisation of Cellulose Fibres

The raw and treated (ATH and AHH) cellulose fibres were sterilised prior to use. The fibres were immersed in filtered 70% ethanol for 30 min on an orbital shaker at 100 rpm, followed by an overnight incubation in fresh 70% ethanol. The fibres were then rinsed three times in phosphate buffered saline (PBS, pH 7.4) to remove residual ethanol.

#### 2.5.2. Cytotoxicity Study

The raw and treated fibres were placed in 96-well plates in triplicates at 5 mg per well and incubated in 300 µL/well of RPMI media (Life Technologies, Victoria, Australia) for 24 h at 37 °C in a humidified environment with 5% CO_2_. Incubated media were transferred to fresh wells and 1 × 10^4^ human fibroblasts/well were seeded into the various extracted media. The cells were then cultured for 4 d and cell viability was assessed using the trypan blue exclusion method. Trypan blue (Sigma-Aldrich) stained the dead cells blue while the viable cells remained clear. Cell viability was calculated by the ratio of live cells to the total number of cells per sample, expressed as a percentage.

## 3. Results and Discussion

The hemp hurd biomass (HHB) used for this study comprises 85% of total solids that contains 77% of holocellulose, 4%–5% of lignin, and 3% of ash. The moisture content (14%) in the sample and the composition of hemp hurd were determined using standard National Renewable Energy Laboratory protocol [25]. The bast fibre (outer covering) of the hemp was taken for industrial purpose and the inner core or hurd was used for this study. The inner core basically consists of xylem and phloem. Sonication applied to milled hemp prior to chemical treatment allowed breakage of strong intermolecular bonds in the structure and partially reduced the biomass size. The exposure of sonicated biomass to sodium hydroxide and hydrogen peroxide enabled opening of the structure. The presence of hydrogen peroxide in the solution allows decolouration of the biomass. Earlier studies conducted using alkaline treatment have reported that the porosity of biomass increases as the lignin is removed from the structure [26]. In our previous study, we observed HHB structure opening and tracheids bundles got exposed after alkaline pretreatment [27].

### 3.1. Scanning Electron Microscopy (SEM) Imaging

**Figure 1:**
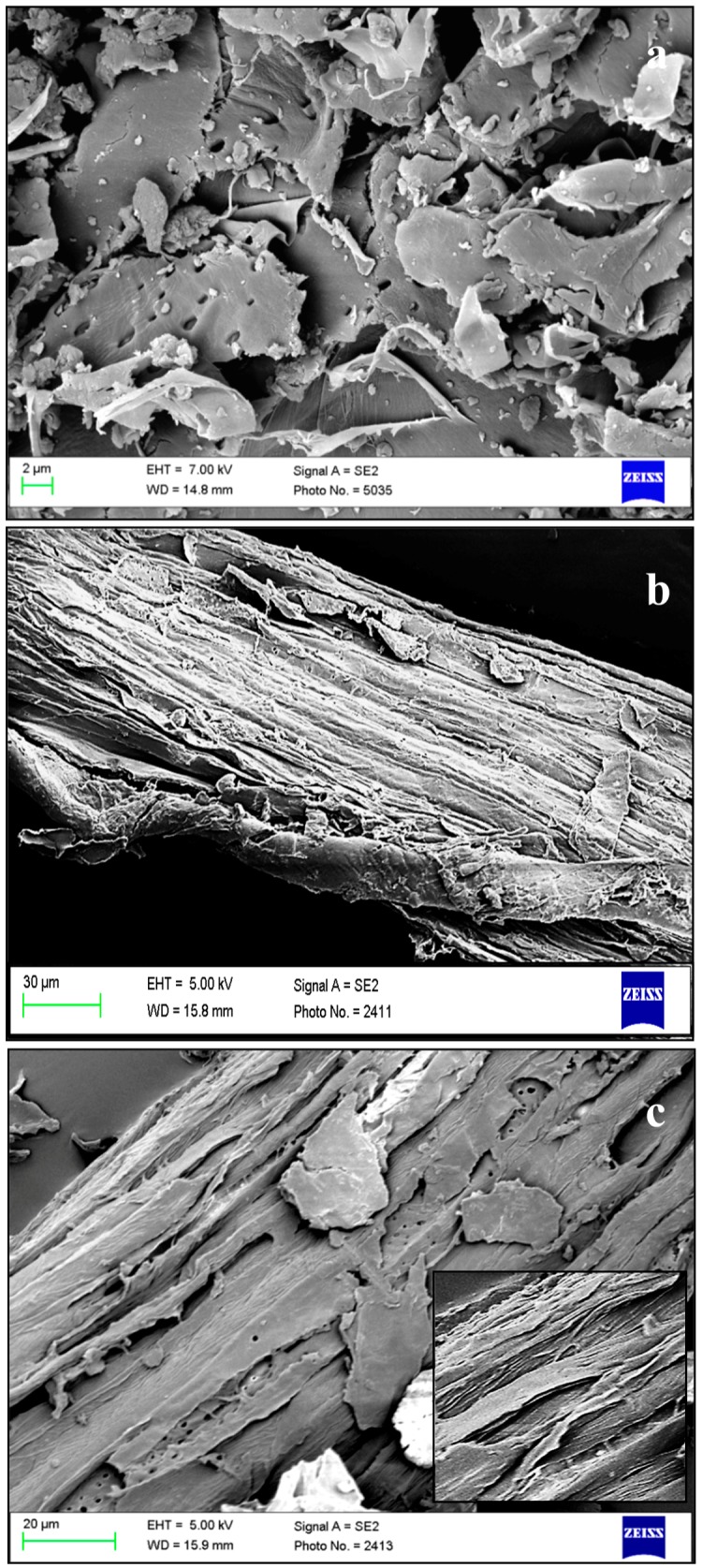
SEM images of HHB at different stages of microfibres extraction: (**a**) raw; (**b**) ATH and (**c**) AHH.

The morphology of raw HHB, ATH and AHH was studied using SEM (Figure 1). The raw HHB appeared to be compact in structure with flakes all over the surface. The alkaline treatment on raw HHB resulted in the partial removal of amorphous part (hemicellulose, lignin) from the surface. These components were responsible in holding the structure tight and rigid. Sonication resulted in the loosening of bonds in the stacked structure and allowed easy penetration of chemicals. The alkaline extraction method did defibrillation and exposed cellulose microfibre bundle all over the surface. The sequential bleaching and acid hydrolysis made these features prominent and resulted in the occurrence of fine cellulose fibres by further removal of amorphous content from the structure. A similar study has reported the appearance of smooth surface with fine fibre indicating effectiveness of sulphuric acid hydrolysis during cellulose whisker formation [28]. Earlier studies have reported that these fibres are a collection of nanofibres linked together with strong hydrogen bonds [29,30]. The strong networking of hydrogen bond forms chiral phase at a certain concentration and retains the structure. Due to the presence of ions, flake formation or flocculation takes place, which hinders the proper penetration creating an uneven distribution of fibres. The formation of such flakes is visible in our studies with holes at the regular interval [31].

### 3.2. Particle Size Analysis

The effect of alkaline and acid treatment on the particle size was analysed using Malvern size particle analyser. The microfibres diameter obtained from the analysis is shown in Table 1. The initial size of the raw HHB powder varied from 300–600 µm. The repeated alkaline treatment reduced the particle size and the diameter from 20–368 µm and about 50% of the particle was in the size range of 114 µm. The diameter further reduced after acid hydrolysis and the particle size ranged between 12–203 µm.

The diameter of hemp microfibres was reduced to 80% (114 µm) after alkaline treatment and 90% (64 µm) after acid hydrolysis from its original size (300–600 µm). The extraction method resulted in loosening of strong bonds. Removal of non-cellulosic content during chemical treatment and appearance of fibres have been reported earlier [32].

### 3.3. ATR-FTIR Studies

**Figure 2:**
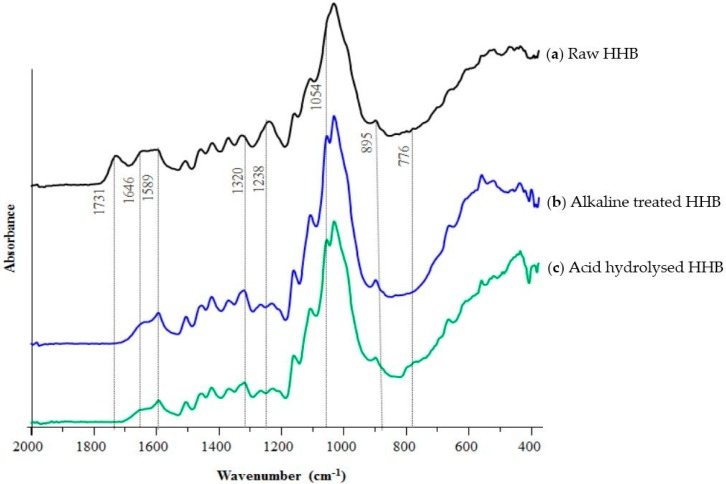
FTIR spectra of HHB at different stages (**a**) raw HHB (black); (**b**) ATH (blue); and (**c**) AHH (green).

The FTIR spectra of raw and extracted cellulose microfibres are represented in Figure 2 and the vibrational attributions are summarised in Table 2. The peak raising around 1731 cm^−1^ in raw hemp represents the acetyl and uronic ester groups arising from hemicellulose or the ester linkage of the carboxylic group from lignin or hemicellulose. The significant disappearance of this peak after alkaline treatment and acid hydrolysis indicates the removal of hemicellulose and lignin [33]. The shouldering around 1646 cm^−1^ attributes to the absorption of water which has also reduced after the treatment in both the samples in comparison to raw biomass [34].

The characteristic peak around 1589 cm^−1^ is due to the C=C stretching vibration of lignin in ATH and AHH samples [35]. The stretching occurring around 1320 cm^−1^ attributes to CH_2_ stretching from cellulose indicating the exposure of cellulose crystalline surface due to the elimination of non-cellulosic components. The significant reduction of peak arising from lignin around 1238 cm^−1^ in extracted microfibres indicates the removal of lignin [36]. The stretching of a peak around 1054 cm^−1^ and 895 cm^−1^ attributes to the C=O stretching and C–H vibration from cellulose. The alkaline treatment affected the height of peak around 1054 cm^−1^ indicating the chemical alteration in the crystalline structure. These observations indicated the successful removal of non-cellulosic components and the opening of cellulose surface.

### 3.4. X-ray Diffraction (XRD) Analysis

**Figure 3:**
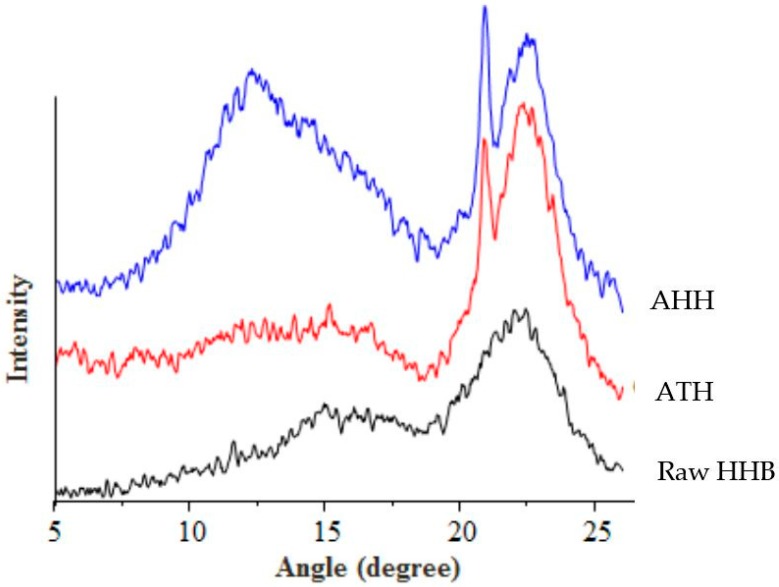
X-ray diffraction pattern of raw HHB (black), ATH (red), and AHH (blue).

The diffractrogram and crystallinity index (calculated by Segal formula) of raw and cellulose extracts are represented in Figure 3 and Table 3. Cellulose exhibits crystalline and amorphous peaks in a X-ray diffractrogram. The amorphous peak occurs around 2θ = 15° and the crystallographic plane representing crystallinity occurs at 2θ = 22°. The raw and extracted microfibres of hemp showed three main peaks at 2θ = 15.4°, 20.8° and 22.4° representing amorphous and crystalline peaks [37].

Due to the alkaline treatment, hemp exhibited polymorphs I and II of cellulose with increased intensity [38]. An increase in the crystallographic plane and a decrease in the amorphous region can be observed after alkaline treatment. The crystallinity index of hemp increased from 39% to 52% after alkali purification. These results supported the data obtained from FTIR suggesting an effective treatment, which destroyed the strong cross-linked structure of biomass.

However, the CrI value of purified samples after acid hydrolysis decreased compared to raw and alkaline treated HHB. As a result of both extraction processes, the chemical structure of biomass got modified by partial removal of amorphous cellulose and lignin. But during the acid purification step, the exposure of crystalline cellulose surface to acid reduced its crystallinity. Previous reports have suggested that the prolonged duration of acid exposure to the purified sample destroys the crystallinity region of cellulose along with the removal of amorphous content [21,33]. As shown in Figure 3, the hydrolysis of alkali treated sample with sulphuric acid for 30 min destroyed the crystallinity of cellulose. Under acidic condition, type II cellulose would have re-precipitated with the extraction time leading to a lower crystallinity peak intensity [39]. However, from the SEM images it can be seen that after acid hydrolysis the occurrence of thin fibres increased and rod shaped fibre bundles have become prominent as seen in Figure 1c. A previously performed crystallinity study on pea fibre showed the occurrence of increased roughness of biomass surface after each purification step, coincided with our observations [40].

### 3.5. Pyrolytic Studies—Thermogravimetric Analysis (TGA)

**Figure 4:**
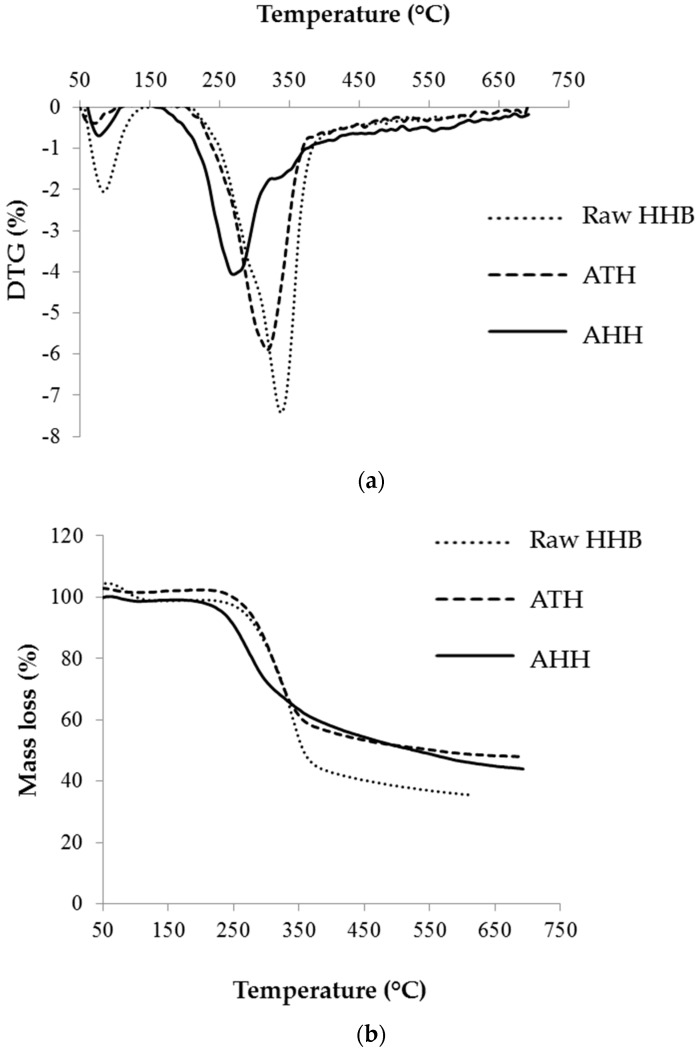
Thermal degradation of raw and treated HHB. (**a**) DTG and (**b**) TG curve.

The pyrolytic behaviour and thermal stability of the raw HHB, ATH and AHH cellulose microfibres were determined by TGA and DSC curve. The thermal degradation, mass loss and onset temperature of all the samples are given in Figure 4 and Table 4. The loss of moisture, carbon dioxide and inorganic compounds initiated after 50 °C and peaked around 100 °C. Peak shift and difference in the intensity of this peak from raw sample to treated samples suggests the removal of water molecules and inorganic components. The major degradation of extracted cellulose was observed between 200 °C–350 °C, whereas raw hemp degraded between 275 °C–350 °C. This indicates the thermal combustion of hemicellulose and cellulose in the raw hemp, which did not occur in purified samples suggesting the removal of non-cellulosic contents (hemicellulose, amorphous cellulose). Previous studies have reported that this pyrolytic behaviour occurred due to the condensation of aromatic rings occurring from lignin and major degradation of cellulose [41]. A shift in the cellulose degradation temperature was observed between ATH and AHH. It has been reported in previous studies that in acid hydrolysis, the cellulose chain becomes even shorter and this tends to lower the degradation temperature [42]. Previous reports have even suggested that such profile can be observed due to the occurrence of sulphate group which lowers the thermostability of samples which was observed in our results also [43].

### 3.6. Enzyme Saccharification

Cellulose microfibres obtained from alkaline and acid hydrolysis were enzyme saccharified using cellulase from *Trichoderma reesei*. The extraction procedure of cellulose enabled partial removal of amorphous (lignin, and pectin) content. This cellulose extract can be a potential source for the enzyme hydrolysis and produce reducing sugars for ethanol fermentation as mentioned in an earlier study [11] and can be taken as an extension of biorefinery perspective.

**Figure 5:**
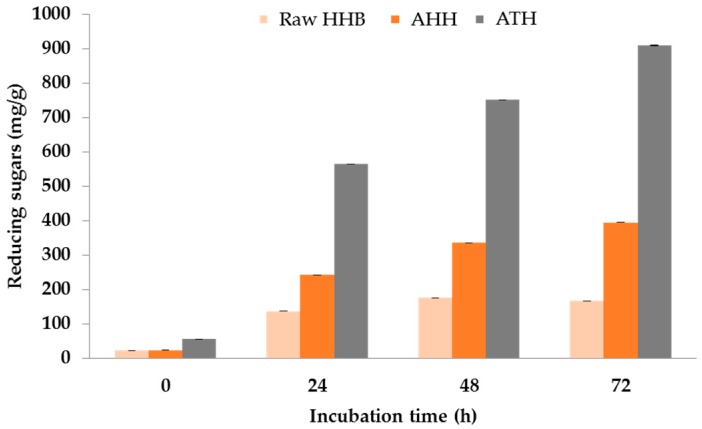
Enzyme saccharification of raw, AHH and ATH for 72 h using cellulase from *Trichoderma reesei*.

The extracted cellulose was digested for 72 h and the resulted reducing sugars are shown in Figure 5. The ATH cellulose exhibited the highest amount of reducing sugars (909 ± 0.02 mg/g) compared to acid hydrolysed (395 ± 0.006 mg/g) and raw biomass (167 ± 0.01 mg/g) in 72 h. This indicates that alkaline treatment produced pure cellulose microfibre bundles by removing lignin and hemicellulose from the structure causing easier access to cellulose and resulted in a high yield of reducing sugars. A suitable method for the use of the hydrolysed sugars for producing omega-3 fatty acids has been developed in a previous study. Our results indicated that these sugars are suitable for growing marine microalgae for producing bioactives [44]. The yield of reducing sugars lowered in samples obtained after sequential alkaline and acid treatment. As mentioned earlier, this could be due to the presence of sulphate group, which destructed the crystallinity of cellulose and potentially lowered the hydrolysis yield.

### 3.7. Toxicity Studies with Human Fibroblasts

**Figure 6:**
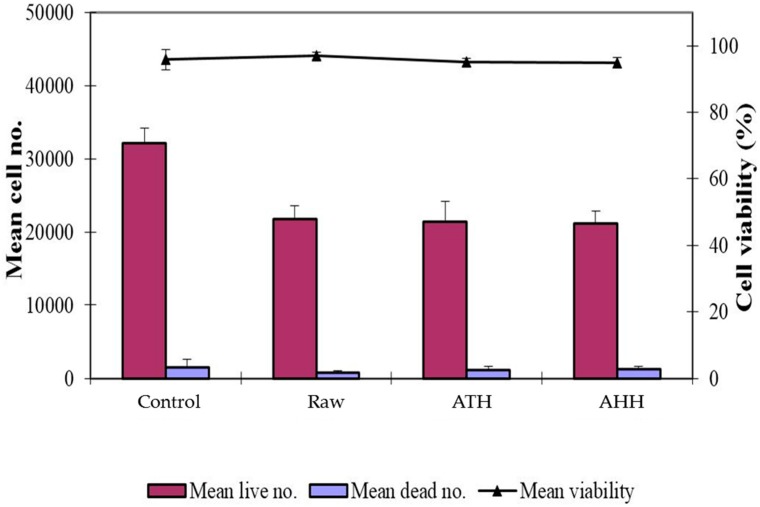
Toxicity study conducted on raw, ATH and AHH for 4 days.

The cellulose microfibres produced by the two different methods, alkaline and acid hydrolysis, were assessed for biocompatibility using human fibroblasts. As shown in Figure 6, there was no difference in cell proliferation (human fibroblasts) between the raw HHB and the treated samples (ATH and AHH). Cell viability greater than 90% was observed across all of the groups indicating that the two extraction methods used to produce cellulose microfibres were not toxic to cells. The excellent biocompatibility of these cellulose extracts lends themselves to be used as biomaterials. Previous studies have demonstrated that various types of cells such as fibroblasts, smooth muscle cells, glioma cells and mesenchymal stem cells attached and proliferated better on micro- and nanostructured surfaces than flat surfaces [45,46]. Studies have demonstrated the usage of cellulose microfibres for tissue engineering, which included tendon/ligament preparation and drug delivery aspect [47]. The ability to use wood waste to produce cost effective nanostuctured natural biomaterials has the potential of generating interest in using cellulosic nanofibres in biomedical applications. Results from this study demonstrated that the bioprocessing method can produce cellulose microfibres that has excellent compatibility, expanding its use to biomedical applications, for example, tissue engineering.

## 4. Conclusions

The extraction of cellulose microfibres from HHB was achieved using alkaline and acid hydrolysis. The characterisation techniques demonstrated that the alkaline treatment majorly worked in the preparation of microfibres and acid hydrolysis tend to shorten the cellulose chain. It produced a higher content of cellulose extract by increasing the crystallinity index from 39% to 52%. Enzyme saccharification of extracted cellulose microfibres was in agreement with characterisation techniques, showing maximum reducing sugar (909 mg/g) yield in alkaline treatment showing its potential application in biofuel production. The toxicity study on human fibroblasts demonstrated both extraction methods as non-toxic with cell viability >90%. The investigation produced non-toxic cellulosic microfibres from hemp biomass, which suggested its possible use in biomedical applications.

## Figures and Tables

**Table 1:** Particle size measurement of ATH and AHH microfibres.

Samples	Microfibres Diameter (µm)		
	d (0.1)	d (0.5)	d (0.9)
Alkaline treatment (ATH)	20.9	114.5	368.7
Acid hydrolysed (AHH)	12.6	64.1	203.0

**Table 2:** Attribution of characteristic peaks of HHB.

Attribution of Characteristic Peak	Wavenumber (cm^−1^)
C=O vibration in hemicellulose and lignin	1731
O–H deformation	1646
Stretching of C=C in aromatic rings of lignin	1589
Deformation of CH_2_ plane in cellulose	1423
CH_2_ stretching in cellulose	1320
C–O wagging in hemicellulose and lignin	1265
C–O stretching of ether in lignin	1238
C–O stretching of hemicellulose and lignin	1054

**Table 3:** Crystallinity index of hemp at different stages of extraction process of HHB.

Samples (Hemp Hurd Biomass)	CrI (%)
Raw Biomass	39
ATH	52
AHH	15

**Table 4:** The onset of pyrolytic degradation and decomposition peak of HHB.

Samples	Onset Degradation	Peak Degradation
	Temperature (T*_o_*)	Temperature (T*_max_*)
Raw HHB	205	337.5
ATH	205	315
AHH	155	265, 327
